# Clinical Outcomes and Factors Associated with Neuroleptic Malignant Syndrome in Older Patients: A Case Control Study

**DOI:** 10.3390/jcm14248901

**Published:** 2025-12-16

**Authors:** Pataraporn Pewloungsawat, Sahaphume Srisuma, Sirasa Ruangritchankul

**Affiliations:** 1 Division of Geriatric Medicine, Department of Medicine, Faculty of Medicine Ramathibodi Hospital, Mahidol University, Bangkok 10400, Thailand; mo_wm@hotmail.com; 2 Division of Clinical Pharmacology and Toxicology, Department of Medicine, Faculty of Medicine Ramathibodi Hospital, Mahidol University, Bangkok 10400, Thailand; boat_ra_ac@hotmail.com

**Keywords:** neuroleptic malignant syndrome, clinical outcomes, older adults, antipsychotics, dopaminergic agents

## Abstract

**Background/Objectives:** Older adults are vulnerable to multiple neuropsychiatric diseases. Antipsychotics and dopamine agents are widely used and are associated with neuroleptic malignant syndrome (NMS). However, NMS has still received limited focus in older Thai adults. This study aimed to evaluate the factors associated with NMS in older Thai adults and to review the clinical manifestations, adverse outcomes, severity, preventability, and management of NMS. **Methods:** The case–control study was conducted on older patients aged ≥ 60 years admitted to Ramathibodi Hospital between 1 August 2008, and 31 July 2022. Data were retrieved from electronic medical record reviews. Patients with NMS were identified by the documented International Statistical Classification of Diseases and Related Health Problems 10th Revision (ICD-10) code G21.0 or ICD-9 code 333.92. The non-NMS or control group was matched by age, sex, time of admission, and agent exposure 14 days before the index date. **Results:** Fifty-four older patients admitted to the hospital were enrolled in this study. Of all, nine (16.7%) were diagnosed to have NMS. The most common causes of NMS were antipsychotic exposure (6 cases). All NMS events were considered to be “preventable” (scores of 4–6) according to the Likert scale. The independent factor associated with NMS was antipsychotic dose > 1 defined daily doses (DDDs) (OR 11.31, 95% CI 1.05–121.84, *p* = 0.045). **Conclusions:** NMS is a preventable condition. Most NMS cases develop from antipsychotic exposure. In addition, a higher daily dose of antipsychotics is associated with the development of NMS.

## 1. Introduction

Worldwide, the geriatric population has markedly increased in recent decades as a result of increased longevity [1]. The global older population aged ≥ 60 years was almost 926 million in 2017 and is predicted to reach 2.1 billion by 2050 [2]. In 2022, Thailand became an aged society, with at least 20% of older adults aged ≥ 60 years. By 2040, the aging Thai population is estimated to increase to 16.7 million [3].

Older people are susceptible to multiple neuropsychiatric diseases, such as dementia with behavioral and psychological symptoms of dementia (BPSD), Parkinson’s disease, delirium, and depression [4]. To treat these diseases and conditions, antipsychotics for behavioral and psychological symptoms in dementia and delirium [5], dopaminergic agents for Parkinson’s disease, and lithium for depression [6] are widely used in clinical practice [7,8]. Neuro-psychopharmacotherapy may be a cause of neuroleptic malignant syndrome (NMS). NMS is a potentially life-threatening idiosyncratic condition with a mortality rate of greater than 30% [9]. The pathophysiology of NMS is explained by three main hypotheses. First, NMS develops from decreased central dopaminergic activity, resulting from dopamine antagonists (antipsychotics) or the abrupt withdrawal of dopaminergic medications [9,10]. The lack of the dopamine neurotransmitter in the thermoregulatory center of the hypothalamus and in the basal ganglia leads to hyperthermia and increased muscle tone, respectively [11]. Second, NMS may arise from the toxic effect of pharmacological agents on calcium regulation in skeletal muscle fibers, resulting in massive calcium influx into muscle cells and sustained muscle contraction (rigidity) with increased temperature [11]. Third, hyperactivity of the sympathoadrenergic system is another hypothesis for the pathophysiology of NMS with autonomic symptoms. Moreover, this mechanism increases intracellular calcium ions in muscle cells, leading to heightened muscle tone and rigidity. In addition, this hypothesis suggests that dopamine receptor blockade in the midbrain–cortex–limbic system pathways may contribute to altered mental status [12]. Therefore, the tetrad of distinctive clinical features of NMS consists of hyperpyrexia (>38 °C), muscular rigidity, altered mental status, and autonomic instability [13] in accordance with the diagnostic for NMS using the Diagnostic and Statistical Manual of Mental Disorders, fifth edition (DSM-5) criteria and Levenson’s criteria [14,15]. Among patients with Parkinson’s disease, NMS usually develops between 18 h and 7 days after the withdrawal of dopaminergic drugs (levodopa and dopamine agonists) [16]. The mechanism of NMS in these patients involves impaired dopaminergic transmission in the lateral hypothalamus as well as central dopaminergic hypofunction in the nigrostriatal pathway and central/peripheral sympathetic discharge [16]. Additionally, alterations in central nervous system neurotransmitters and genetic vulnerability are the mechanisms of NMS. Most antipsychotics are major substrates for CYP450 2D6. The CYP2D6 is a highly polymorphic gene with several variants, resulting in poor and intermediate metabolizers. These variations in the CYP2D6 enzyme affect the metabolism of antipsychotics, potentially leading to increased serum drug concentration and a higher risk for NMS [17,18]. The incidence rate of NMS ranges from 0.02% to 3.2% of patients who were prescribed neuroleptic drugs and increases with advanced age [9]. Older people are prone to develop NMS because of physiological and pharmacokinetic changes, namely decreased drug metabolism and excretion. Moreover, older adults are more susceptible to psychoactive drugs, leading to increased adverse drug outcomes [19,20]. Other risk factors for NMS are dementia, including Alzheimer’s disease (AD), dehydration, genetic polymorphism, and multiple antipsychotic agents with rapid dose escalation [12]. Individuals with AD are at a greater risk of NMS because of the mechanism of dopamine dysfunction, leading to decreased dopamine synthesis and impaired dopamine release [21]. Furthermore, acetylcholinesterase inhibitors for AD treatment contribute to dopamine imbalance and dysfunction as well [22]. To manage NMS, physicians should focus on life-threatening clinical outcomes first and rapidly provide basic or advanced life support with specific and supportive treatments. Specific treatments for NMS include benzodiazepine, bromocriptine, amantadine, and dantrolene, based on the severity of NMS [23,24]. In addition, discontinuing causative agents and eliminating risk factors should be performed [12].

However, NMS in older adults has received limited attention in clinical practice in Thailand. Therefore, one aim of the current study was to identify the factors associated with NMS in older Thai adults. The other study aims were to review the clinical manifestations, adverse clinical outcomes and complications, severity, preventability, and management of NMS in this population.

## 2. Materials and Methods

### 2.1. Materials

We gathered secondary data from electronic medical records of 54 patients who were admitted to wards at Ramathibodi Hospital, Mahidol University between 1 August 2008 to 31 July 2022. To protect confidentiality and privacy, personal information was coded and substituted with a patient identification number and date of birth. All personal confidential data were securely stored in password-protected electronic folders and accessed exclusively by the authorized research team for the purposes of this research. Patient consent was waived due to a secondary retrospective analysis of de-identified data.

In terms of biochemical profiles, all participants completed laboratory investigations. Venous blood samples from all participants were collected with clotted blood, NaF, and EDTA tubes for different target analyses. These serum samples were separated for subsequent analysis at the laboratory at Ramathibodi Hospital, Mahidol University. White blood cell (WBC) count, hemoglobin (Hb), sodium, potassium, calcium, magnesium, phosphorus, uric acid, blood urea nitrogen, creatinine, fasting blood sugar (FBS), aspartate aminotransferase, alanine aminotransferase, serum albumin, creatinine kinase (CK) and thyroid stimulating hormone (TSH) were measured through automated methods (Cobas-Mira, Roche, Milan, Italy).

### 2.2. Methods

#### 2.2.1. Study Design, Setting and Participants

We performed a case–control study in which the data were derived from retrospective electronic medical records (EMRs) of 54 patients aged ≥ 60 years admitted to Ramathibodi Hospital, Mahidol University between 1 August 2008 to 31 July 2022. All eligible patients were expected to have a length of hospital stay (LOS) > 48 h. In addition, patients treated with antipsychotics for psychiatric illnesses, including acute bipolar mania, schizophrenia, and acute psychosis, and for medical conditions such as delirium and dementia with BPSD were included in this study. Patients were excluded if they planned admission or had a terminal illness. The sample size calculation was performed using the OpenEpi sample size application, version 2.3.1. developed by Emory University, Atlanta, GA, USA [25]. The required sample size was calculated according to a recent study on the correlation between increased neuroleptics and NMS [26] with a type I error of 0.05, type II error of 0.1, odds ratio of 38.5, exposed proportion of 0.26, and unexposed proportion of 0.93. Additionally, the number of controls for each case was 5, and the sample size to be calculated was 54. The patients were categorized into two groups: the NMS group and the non-NMS group (control). The patients with NMS were identified by the documented International Statistical Classification of Diseases and Related Health Problems 10th Revision (ICD-10) code of G21.0 or ICD-9 code of 333.92.

The NMS group comprised 9 patients with NMS. The non-NMS group consisted of 45 patients without NMS, who were matched by sex, age (within 5 years), and time of admission (within 3 months of the index cases). Furthermore, the controls were matched by continuous exposure to similar psychotropic drugs in NMS cases 14 days before the index date or with the withdrawal of dopaminergic drugs before the index date.

#### 2.2.2. Data Collection and Measurement Tools

The secondary data of 54 patients who were admitted to wards at Ramathibodi Hospital, Mahidol University between 1 August 2008 and 31 July 2022 (14 years) were provided by EMRs. After ethical approval (7 December 2023), the researchers conducted retrospective chart reviews of baseline characteristics, health services, documented clinical measurements, biochemical profiles, and prescribed medications. In patients with NMS, clinical outcomes and complications, severity, preventability, management, and drugs related to NMS were also collected from EMRs.

##### Baseline Characteristics

The baseline characteristic data included the following: age; sex; marital status; lifestyles, such as smoking, and alcohol drinking; health conditions, such as the primary diagnosis, comorbidities assessed by the Charlson Comorbidity Index (CCI) score, and neuropsychological diseases; and physical status.

##### Healthcare Services

The EMRs also contained healthcare services data, such as LOS and the history of outpatient clinic (OPD) or emergency department visits and hospital admissions within 12 months before admission.

##### Documented Clinical Measurement

We gathered data on documented clinical measurements at the date of admission from EMRs. These data included systolic blood pressure, diastolic blood pressure, pulse rate, respiratory rate, body temperature, and current body weight and body mass index (BMI). BMI was determined by dividing the individual’s weight in kilograms by the square of their height in meters [27].

##### Biochemical Profiles

NMS-associated laboratory results were used for the secondary analysis. Blood sugar, blood urea nitrogen, creatinine, serum sodium, serum potassium, serum calcium, serum magnesium, and serum phosphorus concentrations at the index date (within 3 days where available) were collected from EMRs. Furthermore, data on aspartate aminotransferase, alanine aminotransferase, serum albumin, creatinine kinase, and thyroid-stimulating hormone were gathered.

##### Prescribed Medications and Drugs Related to NMS

Medication lists and the number, name, frequency, route, duration of administration, and doses of all prescribed medications before the occurrence of NMS were recorded. Regarding the causative medications of NMS, the use of dopaminergic-blocking agents, such as antipsychotics, and the withdrawal of dopaminergic agents, such as levodopa, were reported in this study. Additionally, the details of the initial dose (mg/day) and initiation date, any dose modifications (mg/day) and corresponding dates, discontinuation dates, indications for use, and the defined daily dose (DDD) for each medication for the causative agents were identified. This study also collected the newly prescribed medication lists that patients received during the past year.

All prescribed medications were categorized according to the Anatomical Therapeutic Chemical (ATC) codes classification system recommended by the World Health Organization such as N05A [28]. This system refers to the assumed average maintenance dose per day of a drug when used for its primary indication in adults. These standardized values were established by the World Health Organization Collaborating Centre for Drug Statistics Methodology for all medications classified under the ATC system [28]. DDDs serve as a standardized reference for international drug use research for fair comparisons and substitutions across different medications [29]. Therefore, DDDs were applied for an appropriate estimation of antipsychotic dose equivalence. Moreover, drug–drug interactions (DDI) with dopaminergic antagonists were further considered in older patients with NMS, according to the Micromedex Drug Interaction Database [30]. In this study, the interacting medications with antipsychotics, such as acetylcholinesterase inhibitors, lithium, and selective serotonin reuptake inhibitors, were carefully considered as a greater risk of NMS [10,22,31,32].

##### Diagnostic Criteria of NMS

The DSM-5 diagnostic criteria of NMS were used for the re-evaluation of the clinical features of patients with NMS. The DSM-5 criteria for diagnosing NMS are all three major criteria and at least two other criteria [9]. The major DSM-5 diagnostic criteria of NMS are exposure to dopamine antagonists or dopaminergic withdrawal within 72 h before the beginning of symptoms, hyperthermia (>38 °C) measured at least two times orally, and muscle rigidity. Other DSM-5 diagnostic criteria for NMS include altered mental status, diaphoresis, tremor, incontinence, mutism, tachycardia, dysphagia, labile blood pressure, leukocytosis, or elevated CK level [9].

##### Severity and Management of NMS

We evaluated the severity of NMS according to the study of van Rensburg and Decloedt [24]. The severity of NMS was classified as mild, moderate, or severe. Regarding the management of NMS, supportive treatment was the mainstay of treatment, whereas specific pharmacological treatments such as benzodiazepine, bromocriptine, amantadine, and dantrolene were used according to the severity of NMS [24].

##### Adverse Clinical Outcomes of NMS

The clinical adverse consequences of NMS ranged from mild to fatal outcomes. Most common complications of NMS result from severe muscle rigidity, acute renal failure, and rhabdomyolysis. In addition, aspiration pneumonia developed in patients with NMS who had difficulty swallowing and altered consciousness [13,33]. Moreover, sepsis, arrhythmias, myocardial infarction, pulmonary embolism, and cardiopulmonary failure were also reported as adverse outcomes of NMS [34,35].

##### Preventability of NMS

The preventability of NMS was graded using a Likert scale ranging from 1 (definitely non-preventable) to 6 (definitely preventable). Three physicians (one medical toxicologist and two geriatricians) screened all NMS cases. Cases that were considered “not preventable” (score 1) by three reviewers were designated as “non-preventable”. Cases considered to be “preventable” (scores 4–6) were categorized on the basis of the types of issues involved that could be improved, such as those related to diagnosis, treatment, monitoring, or other factors, and were further analyzed for recurring patterns or scenarios [36].

#### 2.2.3. Statistical Analysis

The statistical analyses were performed using the SPSS for Windows Software Package, Version 25 (SPSS Inc., Chicago, IL, USA). The demographic characteristics and clinical and biochemical parameters were shown as the number (percentage) for categorical variables and as the mean ± standard deviation (SD) or median ± interquartile range (IQR) for continuous variables. To compare the data between the NMS and non-NMS (control) groups, Pearson’s chi-square test or Fisher’s exact test was used for categorical variables. Unpaired Student’s t-test or Mann–Whitney U test was performed to compare continuous variables between the two groups. Univariable logistic regression analysis was first used to examine the potentially significant factors for NMS. A multivariable logistic regression analysis was further used to determine the independent risk factors for NMS. The results are reported as odds ratios (ORs) and 95% confidence intervals (CIs). Statistical significance was set at a level of *p* < 0.05. A multicollinearity test was also performed to identify a strong relationship between independent variables. Additionally, subgroup analysis of patients with NMS due to antipsychotic exposure and those due to withdrawal of a dopaminergic agent was also performed.

#### 2.2.4. Ethical Considerations

The research protocol was approved by the Committee on Human Rights Related to Research Involving Human Subjects, Faculty of Medicine Ramathibodi Hospital, Mahidol University (protocol number: COA. No. MURA2023/914) on 7 December 2023. All methods complied with the relevant guidelines and regulations in the Declaration of Helsinki. This case–control study was conducted and reported following the guidelines of the Strengthening the Reporting of Observational Studies in Epidemiology (STROBE) statement. The current study was a secondary analysis of de-identified data provided by the EMR database.

## 3. Results

### 3.1. Baseline Characteristics, and Clinical and Biochemical Profiles

A total of 54 older patients who were admitted to the hospital were enrolled in this study. Of these, nine (16.7%) had NMS. The overall mean age was 70.9 (SD 6.2) years and ranged from 60 to 82 years. A comparison of baseline characteristics and clinical profiles between the groups is shown in Table 1 and Table 2. Most participants were male (*n* = 30; 55.6%) and aged ≥ 70 years (*n* = 30; 55.6%). Three-fourths of the patients were married. The number of patients who frequently visited the OPD within 1 year was significantly higher in the non-NMS group than in the NMS group (*p* < 0.05). In addition, patients with NMS had a significantly longer length of hospital stay than those without NMS [40 days (IQR 13.5–74.50) vs. 3 days (IQR 6–17), *p* = 0.003]. There was no difference in BMI between the two groups (*p* > 0.05). Common comorbidities included hypertension (*n* = 38; 70.4%), dyslipidemia (*n* = 27; 50%), and Parkinson’s disease (*n* = 22; 40.7%). The most common principal diagnosis for antipsychotic prescribing was acute bipolar mania (*n* = 2; 22.2%) in the NMS group and major depressive disorder with psychosis (*n* = 11; 24.4%) in the non-NMS group. There was no difference in the median number of chronic diseases and the CCI score between the two groups.

In terms of prescribed medications, the number of newly prescribed medications within 1 year was significantly higher in the NMS group than in the other group (*p* < 0.001). The median DDDs of antipsychotics in the NMS group was substantially higher than those in the control group (*p* = 0.001), as shown in Table 3. Additionally, patients in the NMS group took anxiolytics, sedatives, or hypnotics more frequently than those in the non-NMS group (*p* = 0.023). There was no substantial difference in the proportion of patients exposed to escalated doses of antipsychotics between the two groups (*p* = 0.121). The biochemical profiles of patients are reported in Supplementary Table S1. There was no significant difference in the biochemical profiles between the two groups.

### 3.2. Factors Associated with NMS

After univariable logistic regression analysis, multivariable logistic regression analysis was performed to determine the factors that are independently associated with NMS. After the multicollinearity test, there was no strong relationship between the independent variables. Table 4, antipsychotic dose >1 DDDs (OR 11.31, 95% CI 1.05–121.84, *p* = 0.045) was independently related to NMS.

In addition, subgroup analysis of patients with NMS due to antipsychotic exposure and those due to withdrawal of a dopaminergic agent was also performed. Thirty-six patients exposed to antipsychotics were categorized into the NMS group and the non-NMS group (Table 5). Of these, six (16.7%) patients developed NMS. Among the patients exposed to antipsychotics, those in the NMS group were more frequently prescribed first-generation antipsychotics and depot administration than those in the non-NMS group (*p* < 0.05). Patients with NMS were more likely to take multiple antipsychotics than those without NMS (*p* = 0.010). The number of patients exposed antipsychotic dose > 1 DDDs in the NMS group were substantially higher than those in the control group (*p* = 0.024). After multivariable adjustment, first-generation antipsychotic use was an independent factor associated with the development of NMS (OR 28.55, 95% CI 1.08–752.93, *p* = 0.045), as shown in Table 6. In the subgroup analysis of patients with NMS due to withdrawal of a dopaminergic agent, there was no independent factor related to developing NMS.

### 3.3. NMS Cases

Nine cases of NMS occurred between 1 August 2008 to 31 July 2022. We re-reviewed the clinical diagnosis and outcomes, associated medications, severity, preventability, and treatment of NMS from the documented EMRs. According to DSM-5 criteria, all major criteria of fever, muscular rigidity, and exposure to causative antidopaminergics within the past 72 h before the beginning of symptoms were present in 100% of the cases (Supplementary Table S2). In three of the nine cases, the temperature reached higher than 40 °C. The average temperature was 38.9 (SD 0.9) °C. The majority (88.9%) of these patients showed autonomic instability, elevated CK concentrations (77.8%), leukocytosis (55.6%), and hyporeflexia (55.6%). Elevated CK levels with a range from 1300 to 12,261 IU/L were reported in seven (77.8%) of the nine patients with NMS. Five patients were admitted to the hospital because of NMS, whereas four patients developed NMS in the hospital.

With respect to drug-related NMS, the common causative agents for NMS were antipsychotics (6 cases), as shown in Table 7. One patient developed NMS due to drug interactions between lithium and haloperidol. Three patients (50%) were treated with combinations of two or more antipsychotic agents administered via both oral and injectable routes. However, no patient who received only injectable antipsychotics developed NMS in this study. Most patients in the study developed NMS from the combined use of typical and atypical antipsychotics. Only one patient developed NMS from a single typical antipsychotic, whereas two patients with NMS had been treated with only atypical antipsychotics. Daily doses of antipsychotics at NMS events were reported as DDDs. The median DDDs of the causative antipsychotics were 1.1 (IQR 0.4–2.4). Half of the NMS cases due to antipsychotic use were exposed to >1 DDDs of causative antipsychotics. Most NMS patients were exposed to the causative antipsychotics within 72 h before the beginning of symptoms. Moreover, we found that three patients developed NMS as a result of the withdrawal of dopaminergic agents (anti-Parkinsonian medications). One patient developed NMS within 24 h after the withdrawal of dopaminergic agents.

The most common severity of NMS was moderate. All NMS events were considered “preventable” according to the Likert scale. Common adverse clinical outcomes and complications of NMS in older adults were acute kidney injury (55.6%), followed by infection (55.6%), and acute hepatitis (44.4%). Three patients with NMS developed rhabdomyolysis or respiratory failure. Only one patient died because of bacterial pneumonia.

Supplementary Table S3 shows all patients with NMS who received both specific and non-specific treatments. Regarding specific treatments, all patients were prescribed bromocriptine, and 70% of them were provided with benzodiazepines. Dantrolene and amantadine were not used for treatment in this study. None of the nine patients received electroconvulsive therapy. Common supportive treatments were hydration (100%), a reduced temperature (100%), oxygen supplement (66.7%), antihypertensives (55.6%), and intubation and a ventilator (33.3%). The clinical characteristics of each patient with NMS are shown in Supplementary Table S4. Only one patient died after being diagnosed with NMS because of pneumonia, resulting in respiratory failure and septic shock after recovery from NMS.

## 4. Discussion

This is the first study to examine the factors associated with NMS in older adults in Thailand. In addition, we also reviewed the etiology, clinical manifestations, causative agents, severity, preventability, complications, and management of NMS in older Thai adults. This study demonstrated that the use of antipsychotic doses > 1 DDDs was associated with an increased risk of developing NMS (OR 11.31, 95% CI 1.05–121.84, *p* = 0.045), aligning with previous evidence indicating that larger dose escalations are linked to a heightened risk of NMS [34,37,38]. Administration of high-dose antipsychotics leads to substantial blockade of dopamine D2 receptors across the nigrostriatal, hypothalamic, mesolimbic, and mesocortical pathways, resulting in a sudden reduction in central dopaminergic activity [11,33]. In addition, a greater antipsychotic exposure has been implicated in enhanced calcium release from the sarcoplasmic reticulum of muscle cells, which contributes to the development of muscle rigidity and hyperthermia characteristics of NMS [11].

Among the NMS cases in this study, more than half of the patients developed NMS within 72 h after initiating neuroleptics or withdrawing dopaminergic agents, aligning with previous studies [39,40]. The most commonly reported symptoms of NMS are dehydration and hyperthermia, while alteration of consciousness and autonomic dysfunction are early manifestations [39]. In the study, NMS was diagnosed on the basis of dopamine blocker exposures or withdrawal from dopaminergic medications within 72 h, muscle rigidity, and hyperthermia, and at least two of the other criteria following the DSM-5 diagnostic criteria of NMS [9]. Many patients in our study had NMS without high CK concentrations, similar to findings’ previous studies [41,42]. Therefore, NMS cases should be considered and treated early, even in the absence of increased CK levels [43]. The mortality rate of NMS in older adults has been reported to be up to 10% [33,44,45] owing to respiratory failure, and renal and cardiac complications, which is consistent with our study’s results. The prognosis and management of NMS are based on its severity. Most patients treated with specific management, such as bromocriptine, dantrolene, or amantadine, had more severe symptoms than those treated with only supportive management, including hydration and cooling down body temperature [44].

In patients who received antipsychotics, we found that patients with NMS were more frequently prescribed first-generation antipsychotics than second-generation antipsychotics, which is consistent with previous studies [9,40]. Besides the types of antipsychotics, the use of depot preparations and polytherapy of neuroleptics, and the concomitant use of lithium or antidepressants are also provocative factors for NMS [10,31,32,46]. In our study, three patients with NMS received multiple oral and depot preparations of antipsychotics, and one simultaneously received antipsychotics with lithium. Similarly, patients who received rapidly increased doses of neuroleptics were prone to NMS, as observed in previous studies [10,13,26]. Parenteral and intramuscular neuroleptics have greater bioavailability than an equivalent oral dose, leading to the development of NMS. Lithium has a greater risk of NMS through a sensitizing effect, resulting in a hypodopaminergic state of the brain [47].

### 4.1. Strengths and Limitations

To the best of our knowledge, the main strength of our study is one of the first investigations for a comprehensive assessment of NMS, including etiology, risk factors, clinical manifestations, severity, preventability, complications, and management in older Thai adults. In addition, this study identified the causative agents of NMS from various points of view, such as type, daily dose, duration, route of administration, concomitant use, and newly prescribed medications. All prescribed medications were categorized according to the ATC codes as standardized measures. Additionally, DDDs serve as a standardized reference for international drug use research for fair comparisons and substitutions across different medications.

However, several limitations of the study have to be considered. First, the data were sourced from routine EMRs with the benefit of real-world practice. Nevertheless, the data have been recorded for clinical use and could not provide many potential confounders, such as activities of daily living, education, and socioeconomic status. Second, the study had a relatively modest sample size for statistical purposes. Third, the ICD-coding diagnosis of NMS was based on documents from physicians. Therefore, this may have led to the underdiagnosis of NMS, which is a limitation of a retrospective database study. Finally, the case–control study design was suitable for the rare outcome, such as NMS, although it restricted the determination of a temporal relationship with exposures.

### 4.2. Further Research and Implications

The intervention for NMS prevention through assessment tools, including medication lists (type, number, and dose), should be developed. Practitioners should observe clinical manifestations and biochemical profiles after they provide antipsychotics or withdraw dopaminergic agents to detect NMS early. In addition, physicians should avoid depot administration of first-generation antipsychotics and the use of multiple or high doses of neuroleptics in older adults to diminish the incidence of NMS. Therefore, comprehensive medication reviews and medication reconciliations play major roles in the prevention of NMS in older adults. Furthermore, guidelines for NMS prevention should be developed and promoted to encourage physicians and clinical pharmacists to use them in clinical practice, thereby reducing the occurrence of NMS [12,13,48], as shown in Table 8.

## 5. Conclusions

NMS is a preventable and curable condition. Heightened awareness of a higher daily dose of antipsychotics could reduce the occurrence of NMS. Physicians should be aware of depot preparations of high-potency neuroleptics, antipsychotic polytherapy, and the concomitant use of lithium or antidepressants to decrease the risk of NMS. Additionally, clinicians should conduct comprehensive medication reviews and medication reconciliations on a regular basis. Guidelines for NMS prevention should be developed to support physicians and clinical pharmacists in using antipsychotics carefully, regarding dosage, type, and route, and in closely monitoring patients.

## Figures and Tables

**Table 1:** Baseline demographics of hospitalized older patients stratified by the presence of NMS.

Baseline Characteristics	All (*n* = 54)	NMS (*n* =9)	Control (*n* = 45)	*p*-Value
		*n* (%)	*n* (%)	
Age in years, mean (SD)	70.9 (6.2)	70.4 (6.9)	70.9 (6.2)	1.000 ^#^
<70 years	24 (44.4)	4 (44.4)	20 (44.4)	
≥70 years	30 (55.6)	5 (55.6)	25 (55.6)	
Female	24 (44.4)	4 (44.4)	20 (44.4)	1.000 *
**Marital status**				
Single, widowed, or separated	13 (24.1)	3 (33.3)	10 (22.2)	0.670 *
Married	41 (75.9)	6 (66.7)	35 (77.8)	
**Lifestyle**				
Alcohol drinking	6 (11.1)	0 (0)	6 (13.3)	0.020 *
Smoking	10 (18.5)	1 (11.1)	9 (20.0)	0.041 *
**Healthcare services**				
History of OPD visits > 10 within 1 year	37 (68.5)	0 (0.0)	37 (82.2)	<0.001 *
History of hospital admission > 1 within 1 year	14 (25.9)	3 (3.33)	11 (24.4)	0.370 *
History of ER visits > 1 within 1 year	9 (16.7)	3 (3.33)	6 (13.3)	0.090 *
LOS, median (IQR)	7 (3, 29.5)	40 (13.5, 74.5)	3 (6, 17)	0.003 ^+^
**Physical profiles**				
Significant weight loss	5 (9.4)	1 (11.1)	4 (9.1)	1.000 *
Ambulation with assistance	22 (40.7)	4 (44.4)	18 (40.0)	1.000 *
Body mass index (kg/m^2^), mean (SD)	22.8 (4.4)	23.3 (4.2)	22.6 (4.4)	0.670 ^#^

Data are presented as mean (standard deviation), *n* (%), or median (interquartile range); * Chi-square test, ^#^ Student’s *t*-test, ^+^ Mann–Whitney U test. Abbreviations: SD, standard deviation; IQR, interquartile range; ER, emergency room; OPD, outpatient department; LOS, length of stay; kg, kilogram; m, meter; NMS, neuroleptic malignant syndrome. Bold: used for category.

**Table 2:** Clinical characteristics among hospitalized older patients stratified by the presence of NMS.

Baseline Characteristics	All (*n* = 54)	NMS (*n* = 9)	Control (*n* = 45)	*p*-Value
		*n* (%)	*n* (%)	
**Comorbidities**				
No. of chronic diseases, median (IQR)	5.5 (4, 8)	8 (5, 8.5)	5 (4, 7)	0.068 ^+^
CCI, median (IQR)	3 (5, 6)	4 (2.5, 5.5)	5 (3, 6.5)	0.133 ^+^
Dyslipidemia	27 (50.0)	6 (66.7)	21 (46.7)	0.467 *
Hypertension	38 (70.4)	7 (77.8)	31 (68.9)	0.709 *
Diabetes mellitus	19 (35.2)	3 (33.3)	16 (35.6)	1.000 *
Coronary artery disease	9 (16.7)	0 (0.0)	9 (20.0)	0.328 *
Congestive heart failure	2 (3.7)	0 (0.0)	2 (4.4)	1.000 *
Arrhythmia	4 (7.4)	0 (0.0)	4 (8.9)	1.000 *
Dementia	12 (22.2)	4 (44.4)	8 (17.8)	0.098 *
BPSD	10 (18.5)	2 (22.2)	8 (17.8)	0.667 *
Hypothyroidism	5 (9.3)	1 (11.1)	4 (8.9)	1.000 *
Cerebrovascular disease	10 (18.5)	1 (11.1)	9 (20.0)	1.000 *
Parkinson’s disease	22 (40.7)	3 (33.3)	19 (42.2)	0.723 *
Cirrhosis	4 (7.4)	0 (0.0)	4 (8.9)	1.000 *
Chronic kidney disease	11 (20.4)	3 (33.3)	8 (17.8)	0.367 *
Depression	16 (29.6)	4 (44.4)	12 (26.7)	0.425 *
Malignancy	14 (25.9)	2 (22.2)	12 (26.7)	1.000 *
COPD	4 (7.4)	0 (0.0)	4 (8.9)	1.000 *
Obstructive sleep apnea	4 (7.4)	1 (11.1)	3 (6.7)	0.529 *

Data are presented as *n* (%), or median (interquartile range); * Chi-square test, ^+^ Mann–Whitney U test. Abbreviations: CCI, Charlson Comorbidity Index; BPSD, Behavioral and Psychological Symptoms of dementia; IQR, interquartile range; COPD, chronic obstructive pulmonary disease; NMS, neuroleptic malignant syndrome. Bold: used for category.

**Table 3:** Prescribed medications among hospitalized older patients stratified by the presence of NMS.

Baseline Characteristics	All (*n* = 54)	NMS (*n* = 9)	Control (*n* = 45)	*p*-Value
		*n* (%)	*n* (%)	
**Prescribed medications**				
Number of prescribed medications per person, median (IQR)	9 (6.8, 12)	9 (7, 11)	9 (6.5, 12)	0.889 ^+^
Number of newly prescribed medications within 12 months, median (IQR)	1 (0, 2)	3 (2, 4.5)	0 (0, 1.5)	<0.001 ^+^
Polypharmacy	49 (90.7)	8 (88.9)	41 (91.1)	1.000 *
DDDs of antipsychotics, median (IQR)	0.1 (0.1, 0.5)	1.1 (0.5, 2.1)	0.1 (0.1, 0.2)	0.001 ^+^
Antipsychotic dose > 1 DDDs	5 (13.9)	3 (50.0)	2 (6.7)	0.024 *
Escalated doses of antipsychotics	4 (11.1)	2 (33.3)	2 (6.7)	0.121 *
**Prescribed medication according to ATC classes and codes**				
A06 Drugs for constipation	11 (20.4)	0 (0.0)	11 (24.4)	0.178 *
A10 Drug used in diabetes	16 (29.6)	2 (22.2)	14 (31.1)	0.709 *
B01 Antithrombotic agents	25 (46.3)	3 (33.3)	22 (48.9)	0.480 *
C02 Antihypertensives	3 (5.6)	0 (0.0)	3 (6.7)	1.000 *
C03 Diuretics	3 (5.6)	1 (11.1)	2 (4.4)	0.428 *
C07 Beta blocking agents	15 (27.8)	1 (11.1)	14 (31.1)	0.417 *
C08 Calcium channel blockers	23 (42.6)	6 (66.7)	17 (37.8)	0.148 *
C09 Agents acting on the renin-angiotensin system	12 (22.2)	2 (22.2)	10 (22.2)	1.000 *
C10 Lipid modifying agents	31 (57.4)	6 (66.7)	25 (55.6)	0.717 *
N03 Antiepileptics	14 (25.9)	4 (44.4)	10 (22.2)	0.216 *
N04 Anticholinergic agents	6 (11.1)	3 (33.3)	3 (6.7)	0.051 *
N05 Psycholeptics	44 (81.5)	9 (100)	35 (77.8)	0.183 *
N05A Antipsychotics	41 (75.9)	9 (100)	32 (71.1)	0.095 *
N05B-N05C Anxiolytics, sedatives and hypnotics	22 (40.7)	7 (77.8)	15 (33.3)	0.023 *
N06 Psychoanaleptics	30 (55.6)	6 (66.7)	24 (53.3)	0.715 *
N06A Antidepressants	29 (53.7)	6 (66.7)	23 (51.1)	0.480 *
N06D Anti-dementia drugs	6 (11.1)	1 (11.1)	5 (11.1)	1.000 *
R06 Antihistamines for systemic use	2 (3.7)	1 (11.1)	1 (2.2)	0.308 *

Data are presented as *n* (%) or median (interquartile range); * Chi-square test, ^+^ Mann–Whitney U test. Abbreviations: IQR, interquartile range; ATC, Anatomical Therapeutic Chemical; NMS, neuroleptic malignant syndrome; DDDs, defined daily doses. Bold: used for category.

**Table 4:** Multivariable logistic regression model for factors associated with NMS.

Associated Factors	Univariable Model OR (95%CI)	*p*-Value	Multivariable Model OR (95%CI)	*p*-Value
Antipsychotic dose > 1 DDDs	14.00 (1.63–120.09)	0.016	11.31 (1.05–121.84)	0.045
Number of newly prescribed medications within 12 months	1.69 (1.13–2.52)	0.010	1.51 (0.95–2.39)	0.080

Data are presented as odds ratio (95% confidence interval). Abbreviations: OR, odds ratio; CI, confidence interval; NMS, neuroleptic malignant syndrome; DDDs, defined daily doses.

**Table 5:** The medical profiles of hospitalized older adults exposed to antipsychotics.

Baseline Characteristics	All (*n* = 36)	NMS (*n* = 6)	Control (*n* = 30)	*p*-Value
		*n* (%)	*n* (%)	
**Route of administration**				
Depot route	3 (8.3)	3 (50.0)	0 (0.0)	0.003 *
Oral route	36 (100.0)	6 (100.0)	30 (100.0)	1.000 *
**Classification of antipsychotics**				
First-generation antipsychotics	6 (16.7)	4 (66.7)	2 (6.7)	0.003 *
Second-generation antipsychotics				
**Antipsychotic dose**				
Antipsychotic dose > 1 DDDs	5 (13.9)	3 (50.0)	2 (6.7)	0.024 *
Antipsychotic use > 1 agents	4 (11.1)	3 (50.0)	1 (3.3)	0.010 *

Data are presented as *n* (%) or median (interquartile range); * Chi-square test. Abbreviations: DDDs, defined daily doses; NMS, neuroleptic malignant syndrome. Bold: used for category.

**Table 6:** Multivariable logistic regression model for factors associated with NMS resulting from antipsychotics.

Associated Factors	Univariable Model OR (95%CI)	*p*-Value	Multivariable Model OR (95%CI)	*p*-Value
Antipsychotic dose > 1 DDDs	14.00 (1.63–120.09)	0.016	0.68 (0.01–38.19)	0.851
First-generation antipsychotic use	28.00 (3.03–258.42)	0.003	28.55 (1.08–752.93)	0.045
Antipsychotic use > 1 agents	29.00 (2.25–373.77)	0.010	3.36(0.09–121.68)	0.508
Number of newly prescribed medications within 12 months	1.52 (1.00–2.31)	0.049	1.47 (0.87–2.47)	0.151

Data are presented as odds ratio (95% confidence interval). Abbreviations: OR, odds ratio; CI, confidence interval; NMS, neuroleptic malignant syndrome; DDDs, defined daily doses.

**Table 7:** The medications associated with NMS in hospitalized older adults.

Characteristics	NMS (*n* = 9) *n* (%)
**Medications associated with NMS**	
No. associated drugs per person, median (IQR)	1 (1,2)
** Dopaminergics (withdrawal)**	**3 (100.0)**
Levodopa/benserazide	2 (66.7)
Levodopa/carbidopa	1 (33.3)
** No. of prescribed antipsychotics**	**6 (100.0)**
1	3 (50.0)
2	2 (33.3)
3	1 (16.7)
** Antipsychotics (agents)**	**10 (100.0)**
Typical antipsychotics	4 (40.0)
Haloperidol (intramuscular)	3 (30.0)
Haloperidol (oral)	1 (10.0)
Atypical antipsychotics	6 (60.0)
Olanzapine (oral)	2 (20.0)
Quetiapine (oral)	3 (30.0)
Paliperidone (oral)	1 (10.0)
Antipsychotics dose at the events, median (IQR)	1.1 (0.4, 2.4)
<0.25 DDDs	0 (0)
0.25–0.49 DDDs	1 (16.7)
0.50–0.74 DDDs	2 (33.3)
>1 DDDs	3 (50.0)
** Admission due to NMS**	**5 (55.6)**

Abbreviations: DDDs, defined daily doses; IQR, interquartile range; NMS, neuroleptic malignant syndrome. Bold: used for category.

**Table 8:** The Guideline for Neuroleptic Malignant Syndrome Prevention.

The Guideline for NMS Prevention
**Pre-prescription assessment**
Identify high-risk patients: dementia, dehydration, prior NMS, CKD, polypharmacy
Review drug interaction with antipsychotics: lithium, antidepressants (SSRI, SNRI)
Evaluate clinical baseline: consciousness, vital signs, renal and hepatic function
**Initiation strategy**
Start with low doses of antipsychotics
Avoid rapid dose escalation and high doses of antipsychotics
Avoid parenteral high-potency antipsychotics such as IM haloperidol
Consider initiating with second-generation antipsychotics
**Early monitoring**
Daily check: temperature, muscular rigidity, mental status, BP, HR, hydration
Immediately stop: fever, rigidity, mental status change, autonomic instability
**Maintenance and long-term prevention**
Maintain the lowest effective dose
Avoid polypharmacy and unnecessary medication
Ensure adequate hydration
Educate medical staff, patients, and caregivers on early NMS symptoms

Abbreviations: NMS, neuroleptic malignant syndrome; CKD, chronic kidney disease; SSRI, selective serotonin reuptake inhibitor; SNRI, serotonin norepinephrine reuptake inhibitor; IM, intramuscular; BP, blood pressure; HR, heart rate. Bold: used for category.

## Data Availability

The data analyzed in the current study are available from the corresponding author upon reasonable request.

## Supplementary Materials

The following supporting information can be downloaded at https://www.mdpi.com/article/10.3390/jcm14248901/s1, Table S1: Biochemical profiles among hospitalized older patients stratified by NMS; Table S2: The clinical diagnosis of NMS in hospitalized older adults; Table S3: The treatment of NMS in hospitalized older adults; Table S4: Case reports of NMS in hospitalized older adults.

## Abbreviations

The following abbreviations are used in this manuscript:

NMS	Neuroleptic Malignant Syndrome
DSM-5	Diagnostic and Statistical Manual of Mental Disorders, fifth edition
EMR	Electronic Medical Record
ICD-10	International Statistical Classification of Diseases and Related Health Problems 10th Revision
BMI	Body Mass Index
LOS	Length of Hospital Stay
OPD	Outpatient Department
CK	Creatinine Kinase
DDDs	Defined Daily Doses
ATC	Anatomical Therapeutic Chemical
DDI	Drug–Drug Interactions
CCI	Charlson Comorbidity Index
