# Supplementary material for: Clinical Outcomes and Factors Associated with Neuroleptic Malignant Syndrome in Older Patients: A Case Control Study
Source: J Clin Med. 2025 Dec 16;14(24):8901. doi: 10.3390/jcm14248901 (PMC12733753; doi:10.3390/jcm14248901)
Supplement: Supplementary file 1 [file jcm-14-08901-s001.zip › Table S1. Biochemical profiles among hospitalized older patients stratified by NMS.pdf]

**Table S1.** Biochemical profiles among hospitalized older patients stratified by the presence of NMS

| Biochemical profiles | All (n= 54) | NMS (n=9) N (%) | Control (n=45) N (%) | P-value |
|---|---|---|---|---|
| White blood cell count (cells/mm$^3$), median (IQR) | 6770 (5430, 9490) | 6800 (6200, 8500) | 6730 (5430, 9825) | 0.943$^+$ |
| Hemoglobin (g/dL), mean (SD) | 11.8 (1.9) | 11.9 (1.9) | 11.7 (1.9) | 0.729$^\#$ |
| Platelet (cell/mm$^3$), median (IQR) | 222500 (178750, 273500) | 241000 (220000, 269000) | 217000 (181000, 273000) | 0.347$^+$ |
| Blood urea nitrogen (mg/dL), median (IQR) | 15 (11, 19) | 17 (10, 20) | 15 (12, 19) | 0.762$^+$ |
| Creatinine (mg/dL), median (IQR) | 0.9 (0.7, 1.1) | 0.9 (0.9, 1.3) | 0.9 (0.7, 1.1) | 0.223$^+$ |
| Sodium (mmol/L), mean (SD) | 139.4 (4.1) | 140.6 (4.4) | 139.2 (4.1) | 0.383$^\#$ |
| Potassium (mmol/L), mean (SD) | 3.9 (0.4) | 3.8 (0.4) | 3.9 (0.4) | 0.586$^\#$ |
| Calcium (mg/dL), mean (SD) | 8.8 (0.6) | 9.1 (0.5) | 8.8 (0.7) | 0.286$^\#$ |
| Magnesium (mg/dL), mean (SD) | 1.9 (0.4) | 2.2 (0.2) | 1.9 (0.4) | 0.058$^\#$ |
| Phosphate (mg/dL), mean (SD) | 3.4 (0.9) | 3.6 (0.6) | 3.4 (0.9) | 0.538$^\#$ |
| Aspartate aminotransferase (U/L), median (IQR) | 28 (21, 38.5) | 29 (22, 40) | 27 (21, 36.5) | 0.551$^+$ |
| Alanine aminotransferase (U/L), median (IQR) | 23 (15, 36.5) | 38 (20, 44) | 23 (15, 33.5) | 0.152$^+$ |
| Alkaline phosphatase (U/L), median (IQR) | 85 (64.8, 124.3) | 72 (65, 99) | 86 (65, 124) | 0.613$^+$ |
| Total bilirubin (mg/dL), median (IQR) | 0.5 (0.4, 0.8) | 0.6 (0.5, 1.1) | 0.5 (0.4, 0.8) | 0.383$^+$ |
| Direct bilirubin (mg/dL), median (IQR) | 0.2 (0.2, 0.3) | 0.3 (0.2, 0.4) | 0.2 (0.2, 0.3) | 0.349$^+$ |
| Albumin (g/L), mean (SD) | 34.3 (5.7) | 35.3 (6.0) | 34.1 (5.6) | 0.567$^\#$ |
| Blood sugar (mg/dL), mean (SD) | 111.6 (20.2) | 105.5 (10.2) | 112.7 (21.4) | 0.361$^\#$ |
| Partial thromboplastin time (sec), mean (SD) | 26.0 (4.2) | 24.6 (3.2) | 26.3 (4.4) | 0.418$^\#$ |
| Prothrombin time (sec), mean (SD) | 12.6 (2.8) | 12.1 (0.8) | 12.7 (2.9) | 0.634$^\#$ |
| Thrombin time (sec), mean (SD) | 14.4 (3.4) | 14.2 (4.4) | 14.5 (3.4) | 0.876$^\#$ |
| Thyroid Stimulating Hormone (uIU/mL), median (IQR) | 1.2 (0.3, 2.1) | 1.1 (0.8, 1.7) | 1.3 (0.2, 2.1) | 1.000$^+$ |
| Free thyroxine (ng/dL), mean (SD) | 1.0 (0.3) | 0.9 (0.1) | 1.0 (0.3) | 0.436$^\#$ |

**Data are presented as** mean (standard deviation) or median (interquartile range)

$^\#$ Student's t-test, $^+$ Mann–Whitney U test

**Abbreviations:** mm, millimeter; g, gram; dL, deciliter; mg, milligram; mL, milliliter; mmol, millimole; L, liter; U, unit; uIU, micro international unit; mmol, millimole; SD, standard deviation; IQR, interquartile range; sec, second; ng, nanogram; NMS, neuroleptic malignant syndrome
