# Supplementary material for: Clinical Outcomes and Factors Associated with Neuroleptic Malignant Syndrome in Older Patients: A Case Control Study
Source: J Clin Med. 2025 Dec 16;14(24):8901. doi: 10.3390/jcm14248901 (PMC12733753; doi:10.3390/jcm14248901)
Supplement: Supplementary file 1 [file jcm-14-08901-s001.zip › Table S2. The clinical diagnosis of NMS in hospitalized older adults.pdf]

Table S2. The clinical diagnosis of NMS in hospitalized older adults

Characteristics	NMS (n=9)<br>N (%)
**Clinical diagnosis (DSM-5 criteria)**	
Exposure to dopamine antagonists or dopaminergics (withdrawal) within the past 72 hours	9 (100)
Hyperthermia	9 (100)
38-40 °C	6 (66.7)
>40 °C	3 (33.3)
Rigidity	9 (100)
Mental status alteration	9 (100)
Blood pressure elevation	8 (88.9)
Blood pressure fluctuation	8 (88.9)
Diaphoresis	0 (0)
Urinary incontinence	0 (0)
Tachycardia (Increased HR ≥ 25% above baseline)	9 (100)
Tachypnea (Increased RR ≥ 50% above baseline)	6 (66.7)
CK elevation	7 (77.8)
1000-5000 (U/L)	4 (44.4)
5001-10000 (U/L)	2 (22.2)
>10000 (U/L)	1 (11.1)
Dysphagia	2 (22.2)
Tremor	4 (44.4)
Leukocytosis	5 (55.6)
Reflex	
Hyporeflexia	5 (55.6)
Normoreflexia	2 (22.2)
Hyperreflexia	2 (22.2)
Negative other conditions	9 (100)

Abbreviations: CK, creatine kinase; U, unit; HR, heart rate; h, hour; L, liter; NMS, neuroleptic malignant syndrome; RR, respiratory rate; °C, degree Celsius; DSM-5, The Diagnostic and Statistical Manual of Mental Disorders, Fifth Edition
