# Supplementary material for: Clinical Outcomes and Factors Associated with Neuroleptic Malignant Syndrome in Older Patients: A Case Control Study
Source: J Clin Med. 2025 Dec 16;14(24):8901. doi: 10.3390/jcm14248901 (PMC12733753; doi:10.3390/jcm14248901)
Supplement: Supplementary file 1 [file jcm-14-08901-s001.zip › Table S3. The treatment of NMS in hospitalized older adults.pdf]

Table S3. The treatment of NMS in hospitalized older adults

Characteristics	NMS (n=9)<br>N (%)
**Specific treatment**	
Bromocriptine	9 (100)
Benzodiazepine	7 (77.8)
**Non-specific treatment**	
Hydration	9 (100)
Oxygen supplement	6 (66.7)
Intubation and ventilator	3 (33.3)
Blood transfusion	3 (33.3)
Antihypertensives	5 (55.6)
Sodium bicarbonate	4 (44.4)
Insulin	3 (33.3)
Intravenous glucose	2 (22.2)
Reduced temperature	9 (100)
Diuretics	4 (44.4)
Nasogastric tube	6 (66.7)

Abbreviations: NMS, neuroleptic malignant syndrome
