# Supplementary material for: Clinical Outcomes and Factors Associated with Neuroleptic Malignant Syndrome in Older Patients: A Case Control Study
Source: J Clin Med. 2025 Dec 16;14(24):8901. doi: 10.3390/jcm14248901 (PMC12733753; doi:10.3390/jcm14248901)
Supplement: Supplementary file 1 [file jcm-14-08901-s001.zip › Table S4. Case reports of NMS in hospitalized older adults.pdf]

Table S4. Case reports of NMS in hospitalized older adults

Case	Age (y) Sex	Causative drugs (duration of treatment)	Indication for drugs associated with NMS	Clinical feature	Treatment	Adverse outcomes	LOS (days)
1.	66; M	Stop Levodopa/carbidopa 125 mg po bid (unknown duration)	Parkinson's disease	T 40; rigidity; tremor; drowsiness; increased BP, RR, HR; labile BP; increased CK	Bromocriptine; lorazepam; sodium bicarbonate; insulin	AKI; rhabdomyolysis; acute hepatitis	9
2.	71; M	Haloperidol 2.5 mg IM bid (1 day)<br>Quetiapine 37.5 mg po (5 days)	BPSD	T 38; rigidity; hyporeflexia; drowsiness; increased BP, RR, HR; labile BP; increased CK	Bromocriptine; lorazepam; sodium bicarbonate	AKI; arrhythmia	39
3.	78; M	Olanzapine 2.5 mg po (9 days)	BPSD	T 38.4; rigidity; drowsiness; increased BP, RR, HR; labile BP; increased CK, WBC	Bromocriptine; antihypertensives; ventilators	Acute respiratory failure; infection (UTI); hypotension	18
4.	78; F	Stop Levodopa/benserazide 125 mg po tid (24 days)	PDD with BPSD	T 40; rigidity; tremor; hyporeflexia; drowsiness; increased BP, RR, HR; labile BP; increased WBC	Bromocriptine; diazepam; ventilators; blood transfusion; intravenous glucose; diuretic	Acute respiratory failure; acute hepatitis; infection (bacterial pneumonia); **Death**	62
5.	62; M	Haloperidol 5 mg po (1 day)<br>Lithium 600 mg po (co-medication)<br>Major drug interaction induced NMS	Bipolar disorder with psychotic episode	T 39.4; hyporeflexia; drowsiness; increased BP, HR; labile BP; increased CK, WBC	Bromocriptine; lorazepam; sodium bicarbonate; insulin; antihypertensives	AKI; rhabdomyolysis; Infection (UTI)	87
6.	60; F	Paliperidone 3 mg po (13 days)	Delusion disorder	T 38; rigidity; drowsiness; increased BP, HR; labile BP; increased CK	Bromocriptine; lorazepam; diazepam	Rhabdomyolysis; acute hepatitis	5
7.	78; F	Stop Levodopa/benserazide 250 mg po (1 day)	Parkinson's disease	T 38; rigidity; drowsiness; tremor; hyporeflexia; increased BP, HR, RR; labile BP; increased WBC	Bromocriptine; blood transfusion; antihypertensives; sodium bicarbonate; diuretic	AKI; Infection (UTI); Metabolic acidosis	90
8.	69; M	Haloperidol 5 mg IM bid<br>Quetiapine 25 mg po bid<br>Olanzapine 5 mg po (1 day)<br>Major drug interaction induced NMS (Haloperidol+Olanzapine)	Bipolar disorder with psychotic episode	T 38.6; rigidity; drowsiness; hyporeflexia; increased HR; increased CK; dysphagia	Bromocriptine; lorazepam; antihypertensives; diuretic	-	53
9.	72; F	Haloperidol 10 mg IM<br>Quetiapine 25 mg po (1 day)	Acute psychosis	T 40; rigidity; tremor; hyporeflexia; drowsiness; increased BP, RR, HR; labile BP; increased CK, WBC	Bromocriptine; lorazepam; blood transfusion; antihypertensives; ventilators; insulin, intravenous glucose; diuretic	Acute respiratory failure; infection (UTI); acute hepatitis; AKI; seizure	40

Abbreviations: M, male; F, female; y, year; mg, milligram, IM, intramuscular; po, per oral; bid, bis in die; tid, ter in die; NMS, neuroleptic malignant syndrome; BPSD, behavioral and Psychological Symptoms of Dementia; T, temperature; BP, blood pressure; RR, respiratory rate; HR, heart rate; CK, creatine kinase; WBC, white blood cell; AKI, acute kidney injury; UTI, urinary tract infection; LOS, length of stay
